# Association of *MUC19* Mutation With Clinical Benefits of Anti-PD-1 Inhibitors in Non-small Cell Lung Cancer

**DOI:** 10.3389/fonc.2021.596542

**Published:** 2021-03-22

**Authors:** Li Zhou, Litang Huang, Qiuli Xu, Yanling Lv, Zimu Wang, Ping Zhan, Hedong Han, Yang Shao, Dang Lin, Tangfeng Lv, Yong Song

**Affiliations:** ^1^Department of Respiratory and Critical Care Medicine, Affiliated Jinling Hospital, Medical School of Nanjing University, Nanjing, China; ^2^Department of Respiratory and Critical Care Medicine, Affiliated Jinling Hospital, School of Medicine, Southeast University, Nanjing, China; ^3^Department of Respiratory and Critical Care Medicine, The Second Hospital of Nanjing, Nanjing University of Chinese Medicine, Nanjing, China; ^4^Geneseeq Technology Inc., Nanjing, China; ^5^Department of Respiratory and Critical Care Medicine, The Affiliated Suzhou Hospital of Nanjing Medical University, Suzhou, China

**Keywords:** *MUC19* mutation, predictive biomarker, whole exome sequencing, immunotherapy, lung cancer

## Abstract

Although anti-PD-1 inhibitors exhibit impressive clinical results in non-small cell lung cancer (NSCLC) cases, a substantial percentage of patients do not respond to this treatment. Moreover, the current recommended biomarkers are not perfect. Therefore, it is essential to discover novel molecular determinants of responses to anti-PD-1 inhibitors. We performed Whole Exome Sequencing (WES) in a cohort of 33 Chinese NSCLC patients. Patients were classified into the durable clinical benefit (DCB) and no durable benefit (NDB) groups. Infiltrating CD8^+^ cells in the tumor microenvironment (TME) were investigated by immunohistochemistry. We also used public datasets to validate our results. In our cohort, good clinical responses to anti-PD-1 inhibitors were more pronounced in younger patients with lower Eastern Cooperative Oncology Group (ECOG) scores and only extra-pulmonary metastasis. More importantly, we identified a novel *MUC19* mutation, which was significantly enriched in DCB patients (*P* = 0.015), and *MUC19-*mutated patients had a longer progression-free survival (PFS) (hazard ratio = 0.3, 95% CI 0.1–0.9; *P* = 0.026). Immunohistochemistry results indicated that the *MUC19* mutation was associated with increased infiltration by CD8^+^ T cells in the TME (*P* = 0.0313). When combining *MUC19* mutation with ECOG scores and intra-pulmonary metastasis status, patients with more positive predictors had longer PFS (*P* = 0.003). Furthermore, *MUC19* mutation was involved in immune responses and associated with a longer PFS in the Memorial Sloan-Kettering Cancer Center (MSKCC) cohort. Collectively, we identified that *MUC19* mutations were involved in immune responses, and NSCLC tumors harboring mutated *MUC19* exhibited good responses to anti-PD-1 inhibitors.

## Introduction

The PD-1/PD-L1 blockade, which reactivates the anti-tumor activity of CD8^+^ T cells by blocking T cell signals, has dramatically revolutionized the management of non-small cell lung cancer (NSCLC) over the past decade (1). Although treatment with anti-PD-1 inhibitors has demonstrated impressive response rates and durable disease remission (2), only a small subset of patients can benefit from them (3). Currently, anti-PD-1 inhibitors that have been approved or are in clinical research include pembrolizumab, nivolumab, atezolizumab, toripalimab, and sintilimab. Apart from their high efficacy, these drugs also display significant immunotoxicity in clinical practice (4), and the cost is high. Therefore, identifying which patients might most likely derive clinical benefit from PD-1/PD-L1 blockade is an essential challenge to be resolved (5). Thus, effective biomarkers for predicting PD-1/PD-L1 inhibitor efficacy are urgently needed in clinical practice.

PD-L1 expression is the earliest and most widely used predictive biomarker for PD-1/PD-L1 inhibitors (6), but it is limited by the detection technology employed (multiple detection antibodies, instrument platforms, different thresholds for positivity) and histological sources of PD-L1 (immune and tumor cells, primary and metastatic tumor sites, and dynamic changes in PD-L1 after treatment) (7). Consequently, additional biomarkers, including microsatellite instability (8) and tumor mutational burden (TMB) (3), have been evaluated. Recently, TMB has also been approved by the Food and Drug Administration as a new predictive biomarker for patients with unresectable or metastatic solid tumors receiving pembrolizumab (9). Nevertheless, similar to PD-L1 expression, TMB is not perfectly correlated with immunotherapy responses, with only a 30–50% objective response rate for TMB-high patients (10). An increasing number of studies have suggested other potential biomarkers, including somatic mutations in specific genes (11, 12), copy number alterations affecting immune-related genes (13), tumor infiltrating lymphocytes (14), and inflamed gene expression profiles (15, 16). Therefore, identification of additional novel biomarkers or combining different biomarkers with greater predictive values is crucial for stratifying populations potentially benefiting from immunotherapy (17).

In this context, we performed Whole Exome Sequencing (WES) to explore and uncover novel molecular determinants of anti-PD-1 inhibitors. In order to explore the underlying mechanisms, we detected CD8^+^ T cells by immunohistochemistry. *MUC19* mutation was associated with good responses to anti-PD-1 inhibitors. These results were further validated in public datasets, encompassing lung cancer patients receiving immunotherapy with *MUC19* mutation data, which further confirmed the association of *MUC19* mutation with good efficacy of anti-PD-1 inhibitors.

## Materials and Methods

### Patient Recruitment and Sample Collection

**Figure 1:**
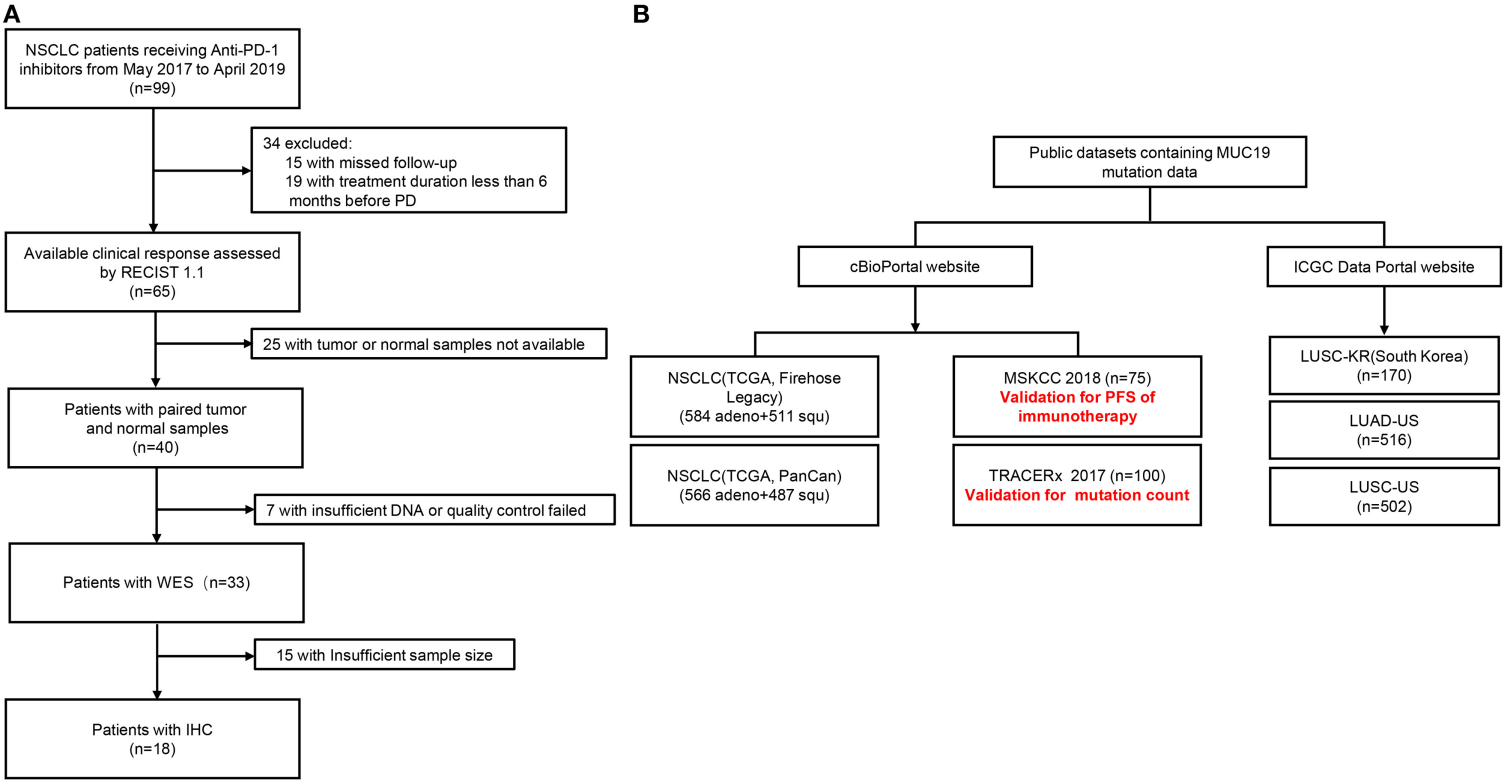
Patient flow of our cohort and public datasets. **(A)** Patient flow of our cohort. **(B)** Patient flow of public datasets.

A total of 99 NSCLC patients receiving anti-PD-1 inhibitors at the Department of Respiratory and Critical Care Medicine of the Affiliated Jinling Hospital, Medical School of Nanjing University, between May 19, 2017, and April 26, 2019, were enrolled. Among them, we were able to assess efficacy in 65 patients using Response Evaluation Criteria In Solid Tumors (version.1.1). The clinical benefits of anti-PD-1 inhibitors were defined as durable clinical benefit (DCB: complete response, partial response, or stable disease lasting > 6 months) and no durable clinical benefit (NDB: progression disease or stable disease that lasted ≤ 6 months). Body mass index (BMI) was calculated as weight in kilograms divided by height in meters squared. WES was performed in 33 patients who could be defined as DCB and NDB and had tumor tissue/matched control samples prior to immunotherapy (Figure 1A). The time from the beginning of immunotherapy to the date of disease progression was defined as progression-free survival (PFS). The study was approved by the Ethical Review Committee of the Affiliated Jinling Hospital and all patients had signed informed consent. The clinical characteristics of the 33 patients were presented in Table 1.

**Table 1:** Baseline clinical characteristics of NSCLC patients in our cohort.

**Characteristics**	**Total (*N* = 33)**	**%**
**Age (years), median range**	64	(36–83)
**<** 65	18	54.5
≥ 65	15	45.5
**Sex**		
Male	25	75.8
Female	8	24.2
**Performance status**		
0–1	25	75.8
≥ 2	8	24.2
**Smoking status**		
Former/Current	19	57.6
Never	14	42.4
**Histology**		
Adenocarcinoma	16	48.5
Squamous cell carcinoma	15	45.5
Other	2	6.1
**Clinical benefit**		
DCB	20	60.6
NDB	13	39.4
**Actionable drivers**		
Yes	8	24.2
- *EGFR* mutation	4	
- *ALK* rearrangement	1	
No	25	75.8
**Stage**		
III	10	30.3
IV	23	69.7
**Metastasis site**		
Lymph node (yes/no)	25/8	75.8/24.2
Lung (yes/no)	14/19	42.4/57.6
Bone (yes/no)	9/24	27.3/72.7
Liver (yes/no)	2/31	6.1/93.9
Brain (yes/no)	6/27	18.2/81.8
Adrenal (yes/no)	4/29	12.1/87.9
**Previous treatment**		
No prior therapy	11	33.3
Platinum-based chemotherapy	14	42.4
Others	8	24.2
**Immunotherapy regimen**		
PD-1 inhibitors	8	24.2
PD-1 inhibitors + Chemotherapy	25	75.8
**Therapy Line**		
1st	11	33.3
2nd	7	21.2
≥ 3rd	15	45.5

In addition, we also used public datasets (cBioPortal: https://www.cbioportal.org/, and International Cancer Genome Consortium Data Portal: https://dcc.icgc.org/) to validate our results (Figure 1B). Among them, the Memorial Sloan-Kettering Cancer Center (MSKCC) cohort was used to verify the relationship between *MUC19* mutation and response to immune checkpoint inhibitors. Data from the MSKCC cohort (18) were downloaded from the cBioPortal website, which contained WES results of 75 NSCLC patients treated with nivolumab plus ipilimumab.

### WES

Tumor tissues/matched control samples were sent to Geneseeq Inc. (Nanjing, China) for WES. The mean target coverage was 150 × for tumor tissue and 60 × for normal controls.

### CD8 Immunohistochemistry

Four micrometer-thick paraffin-embedded tissue sections were used for CD8 immunohistochemistry. Tissue sections were stained with monoclonal anti-CD8 antibody (clone C8/144B, 70306S) from Cell Signaling Technology. Lymphocytes with membranous staining were regarded as positive for CD8. All immunohistochemical sections were independently evaluated by two pathologists, and all evaluation scores were recorded. Two pathologists independently counted CD8^+^ cells and randomly selected 4–6 fields (200 ×) for each immunohistochemical section.

### Statistical Analysis

Fisher's exact test or Chi-squared test was used to compare clinical parameters and gene mutation status between DCB and NDB patients. Differences in CD8^+^ T cells and TMB were examined using the non-parametric Mann-Whitney U test. The Kaplan-Meier method was used to analyze survival [PFS/overall survival (OS)]. Univariate Cox regression analysis was used to define hazard ratios. SPSS v.23.0 and GraphPad Prism v.6 were used for analysis, and *P* values < 0.05 were considered statistically significant.

## Results

### Clinical Characteristics of Patients in Our Cohort and MSKCC Cohort

**Figure 2:**
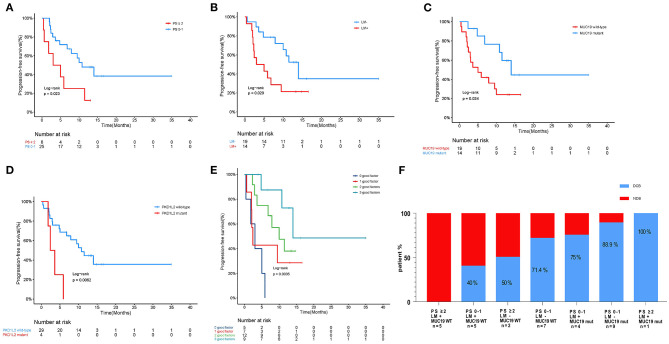
Association of clinicopathological characteristics and gene mutation with anti-PD-1 inhibitor responses in our cohort. **(A)** Kaplan-Meier curves of PFS comparing patients with low performance (PS 0–1) and high performance (PS ≥ 2). **(B)** Kaplan-Meier curves of PFS comparing patients with or without intra-pulmonary metastasis. LM+, intra-pulmonary metastasis in the presence or absence of other extra-pulmonary metastases; LM-, only extra-pulmonary metastasis. **(C)** Kaplan-Meier curves of PFS comparing patients with mutant and wild-type *MUC19*. **(D)** Kaplan-Meier curves of PFS comparing patients with mutant and wild-type *PKD1L2*. **(E)** Effect of *MUC19* mutation status combined with performance and intra-pulmonary metastasis status on PFS in our cohort. **(F)** Histograms depicting proportions of patients who experienced DCB or NDB in different groups, defined by performance status (PS 0–1 or PS ≥ 2), intra-pulmonary metastasis (yes or no), and *MUC19* mutation status (mutant or wild-type), as indicated.

In our cohort, we performed WES in 33 patients who could be defined as DCB and NDB groups and who had tumor tissues/matched control samples prior to immunotherapy. Their clinical characteristics were presented in Table 1. Among them, 18 patients (54.5%) were younger than 65 years, and 25 patients (75.8%) were male. Adenocarcinoma was the most common histology, found in 48.5% of cases, followed by squamous cell carcinoma, found in 45.5% of cases. 42.4% patients had previously received platinum-based chemotherapy, 24.2% patients had previously received TKIs and anti-angiogenesis therapy, and the remaining 33.3% patients had no prior therapy before immunotherapy. The immunotherapy regimens included combination of PD-1 inhibitors and chemotherapy (75.8%), and monotherapy (PD-1 inhibitors, 24.2%). Table 2 showed that good responses were more pronounced in younger patients and those with lower Eastern Cooperative Oncology Group (ECOG) scores, and only extra-pulmonary metastasis. In addition, patients with lower ECOG scores (*P* = 0.023) (Figure 2A) and only extra-pulmonary metastasis exhibited more prolonged PFS (*P* = 0.029) (Figure 2B).

**Table 2:** Associations of anti-PD-1 inhibitor efficacy with clinical characters in our cohort.

**Parameter**	**DCB**	**NDB**	***P* value**
**Age**			0.038
<65	14	4	
≥65	6	9	
**Sex**			0.681
Male	16	9	
Female	4	4	
**Performance status**			0.035
0–1	18	7	
≥ 2	2	6	
**Smoking status**			1.000
Former/Current	12	7	
Never	8	6	
**Histology**			0.393
Adenocarcinoma	8	8	
Squamous cell carcinoma	11	4	
Other	1	1	
**Stage**			1.000
III	6	4	
IV	14	9	
**Metastasis site**			
Lymph node (yes/no)	14/6	11/2	0.432
Lung (yes/no)	5/15	9/4	0.012
Bone (yes/no)	6/14	3/10	1.000
Liver (yes/no)	1/19	1/12	1.000
Brain (yes/no)	5/15	1/12	0.364
Adrenal (yes/no)	3/17	1/12	1.000
**Therapy Line**			0.698
1st	7	4	
2nd	5	2	
≥ 3rd	8	7	
**Treatment**			0.681
Monotherapy	4	4	
Combination therapy	16	9	

In MSKCC cohort, we chose 75 patients who received immunotherapy and who had *MUC19* mutation data. Their clinical characteristics are presented in Supplementary Table 1. Among them, 39 (52.0%) patients were younger than 65 years, 37 patients (49.3%) were male, and 16 (21.3%) had squamous cell carcinoma. We also found that a lower ECOG score was significantly correlated with better clinical benefits of anti-PD-1 inhibitor treatment (*P* = 0.0139).

### Association of *MUC19* Mutation With Clinical Benefits of Anti-PD-1 Inhibitors and Infiltration of CD8^+^ T Cells in Our Cohort

**Figure 3:**
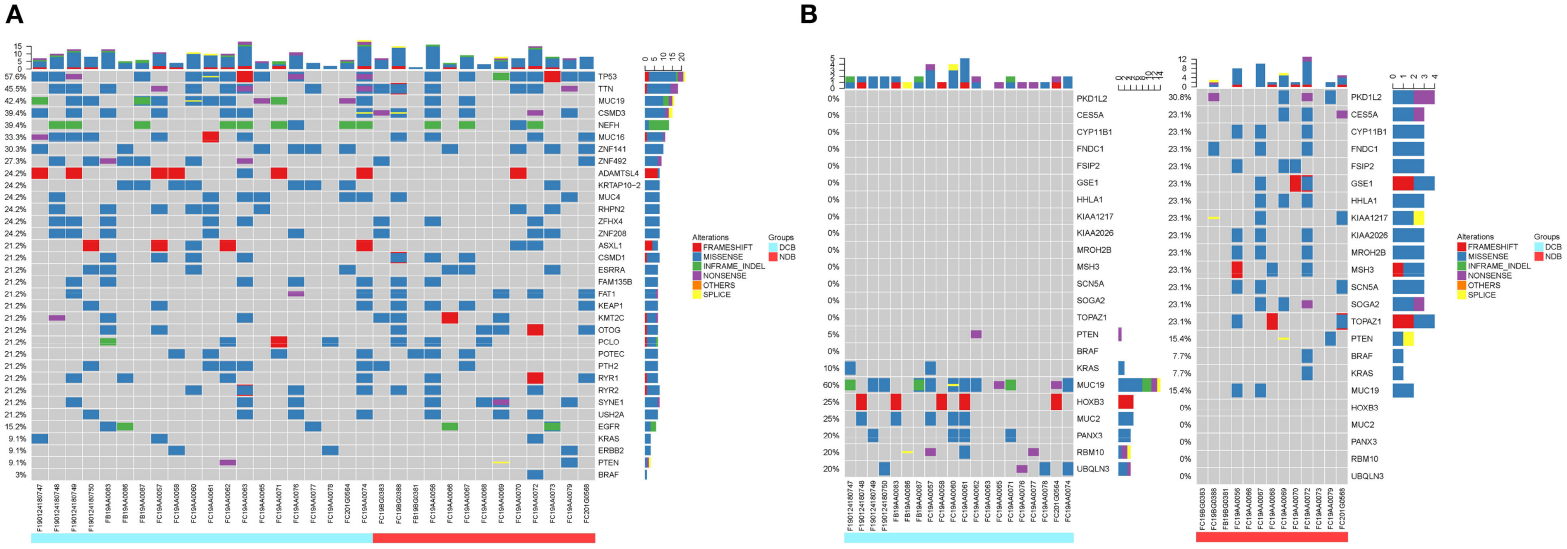
Summary of molecular features associated with anti-PD-1 inhibitor responses. **(A)** The top mutation genes revealed by WES are listed. Sample IDs are shown at the bottom. Mutation frequencies are displayed on the left, and gene abbreviations are listed on the right. Icons representing mutation types are listed in different colors (red = frameshift, blue = missense, green = inframe-indel, purple = nonsense, yellow = splice, and orange = others). The top three mutation genes are *TP53, TTN*, and *MUC19* [71.4% (10/14) missense, 21.4% (3/14) inframe-indel, 14.2% (2/14) nonsense, and 7.14% (1/14) splice]. **(B)** Different high-frequency mutations in patients with DCB (left, blue) and NDB patients (right, red).

To investigate whether individual gene mutations were associated with response or resistance to anti-PD-1 inhibitor treatment, we first focused our analysis on total gene mutations. The top gene mutations in our cohort were shown in Figure 3A; approximately half of the patients harbored a *TP53* mutation (57.6%). In addition to *TP53* mutations, we also found that the mutation rates of *TTN* (45.5%) and *MUC19* (42.4%) were both > 40%. Other common mutations, involving genes such as *EGFR, ERBB2, KRAS, PTEN*, and *BRAF*, were identified in 15.2, 9.1, 9.1, 9.1, and 3% of patients, respectively, and the related percentage was similar to a prior WES study performed in Chinese NSCLC patients (3). We further compared the gene mutations between DCB and NDB patients. Interestingly, we found that there were large differences in high-frequency mutations between the DCB and NDB groups (Figure 3B). Of these, mutations involving *MUC19* (*P* = 0.015) and *PKD1L2* (*P* = 0.017) were significantly enriched in the DCB and NDB groups, respectively. We also found that the mutation rate of *PTEN* (DCB vs. NDB, 5 vs. 15.3%) and *BRAF* (DCB vs. NDB, 0 vs. 7.7%) were higher in the NDB group, while *KRAS* was higher in the DCB group (DCB vs. NDB, 10 vs. 7.7%), which was consistent with previous reports (19), although it did not reach statistical significance, likely owing to small numbers. In addition, we calculated TMB results. Although TMB is a predictive biomarker for the efficacy of immunotherapy recommended by guidelines (9), there were no significant differences involving TMB in our cohort (Supplementary Figure 1).

We next evaluated the association between gene mutations and patient survival. Of all the patients included, 16 died at the time of data collection. The median PFS for all 33 patients was 9.5 months (95% CI 4.5–14.4) and median OS was 26.2 months (95% CI 11.6–40.7). We examined PFS and gene mutations and found that compared with wild-type patients, *MUC19*-mutated patients had significantly longer PFS (*P* = 0.024) (Figure 2C), while *PKD1L2*-mutated patients had a shorter PFS (*P* = 0.006) (Figure 2D). In addition, we also discovered that *FNDC1, FSIP2, GSE1, KIAA1217, LRRK2, OTOGL, SCN5A, SRRT*, and *TOPAZ1* gene mutations were potentially poor prognostic factors for immunotherapy (Supplementary Table 2).

According to the above results, PFS was significantly prolonged in patients with lower ECOG scores, only extra-pulmonary metastasis, and *MUC19* mutation. Each of these variables is important for predicting sensitivity or resistance to immunotherapy; however, each also has limitations in its ability to explain immune checkpoint inhibitor responses. Combining different biomarkers is crucial in stratifying populations benefiting from immunotherapy (17). Therefore, we combined the above variables to test whether this could lead to improved PFS. Intriguingly, when we combined these variables, patients with more positive predictors had longer PFS (Figure 2E). The combination of the three factors together was best in predicting clinical outcomes (Figure 2F).

**Figure 4:**
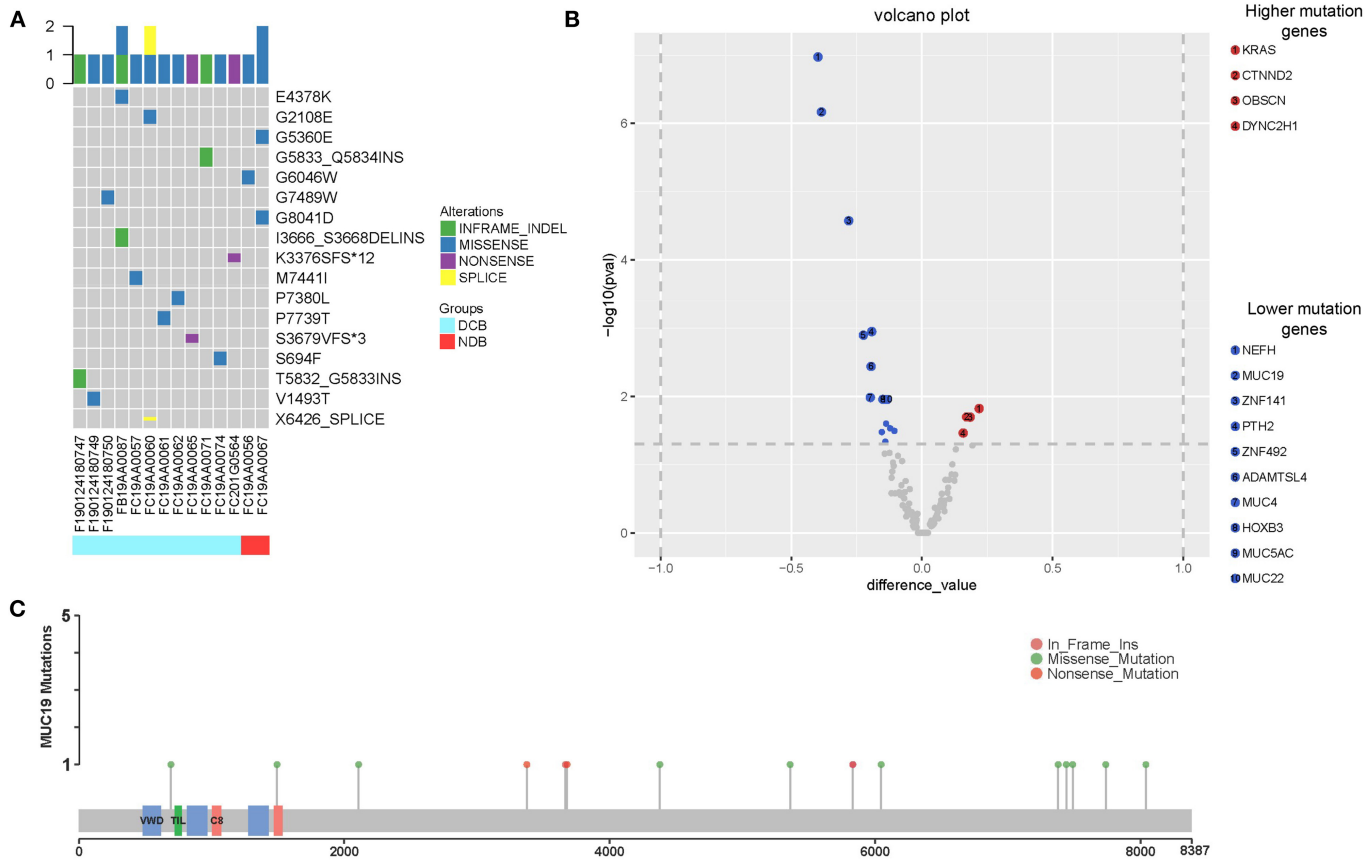
Summary of MUC19 mutation and differential mutated genes between our cohort and MSKCC cohort. **(A)** *MUC19* mutation map of 14 patients including 12 DCB and 2 NDB patients. Sample IDs are shown at the bottom, mutation types on the top and mutants on the right. Icons representing mutation types are listed in different colors (blue = missense, green = inframe-indel, purple = nonsense, yellow = splice). **(B)** Volcano plot displaying differential mutated genes between our cohort and MSKCC cohort. X axis: difference value between gene mutation frequency of our cohort and MSKCC cohort. Y axis: -log10 (pval). Significant events refer to (|difference value| > 0.1 and *p* < 0.05); compared to our cohort, significantly higher mutation genes in MSKCC cohort are in red, significantly lower in blue, others in gray. **(C)** Lolliplot of *MUC19* mutations.

**Figure 5:**
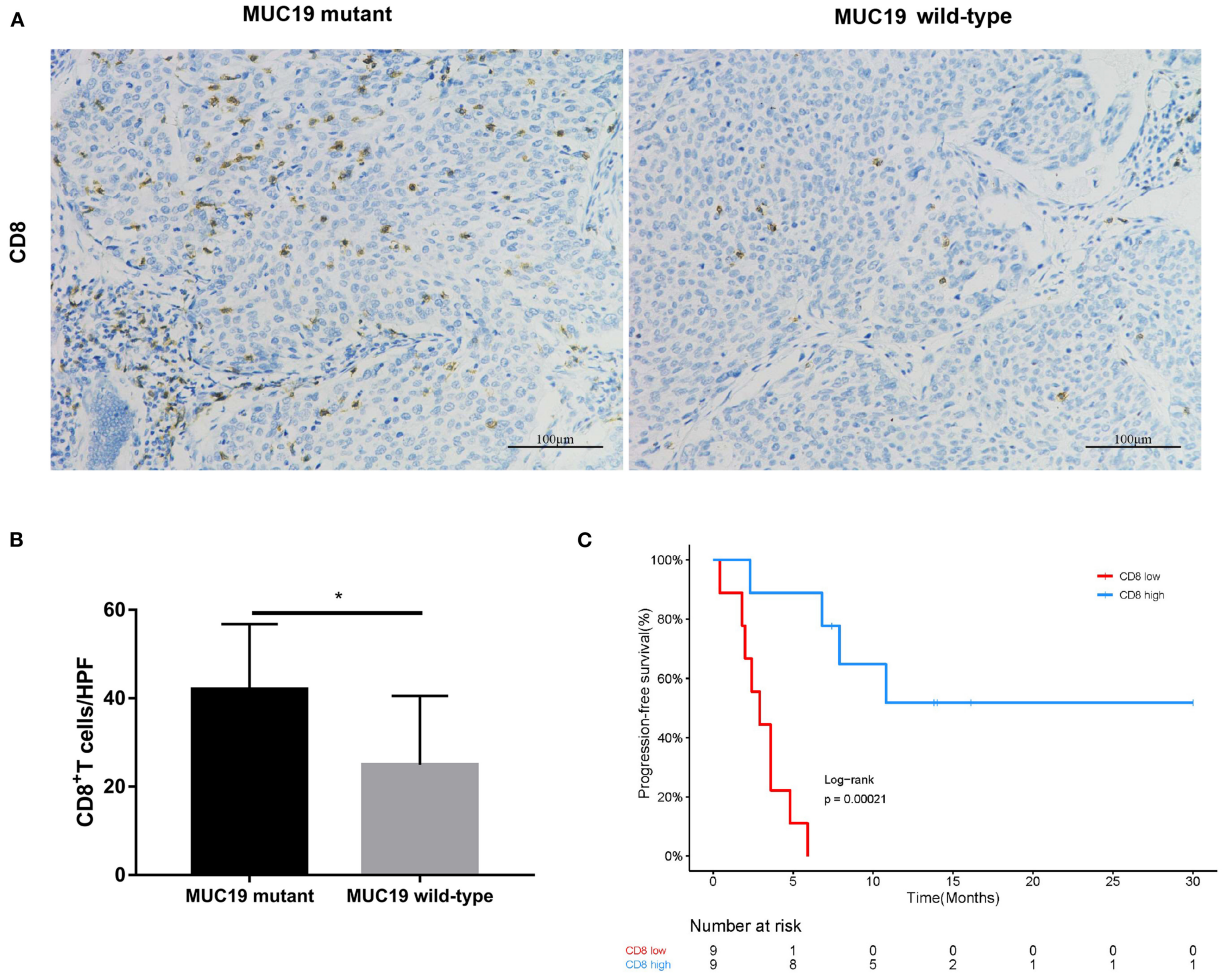
Association of *MUC19* mutation with CD8^+^ T cell infiltration. **(A)** Representative images of CD8 staining in *MUC19*-mutant (left) and wild-type (right) patients. Acquired at × 200 magnification. Scar bar = 100 μm. **(B)** Manually-counted average CD8^+^ cells/HPF are shown in the bar. HPF: high-power field. **(C)** Kaplan-Meier curves of PFS comparing patients with high and low CD8. *P < 0.05.

In our study, there were 14 patients with *MUC19* mutation (Figures 4A,C). Among them, 71.4% (10/14) were missense, 21.4% (3/14) were inframe-indel, 14.2% (2/14) were nonsense, and 7.14% (1/14) were splice; 2 patients had two types of *MUC19* mutation. In DCB patients (12 patients), the mutation types were missense, inframe-indel, nonsense and splice; in NDB patients (2 patients), the mutation type was missense. In addition, the *MUC19* mutants (E4378K, G2108E, G5360E, G5833_Q5834INS, G6046W, G7489W, G8041D, I3666_S3668DELINS, K3376SFS^*^12, M7441I, P7380L, P77380L, P7739T, S3679VFS^*^3, S694F, T5832_G5833INS, V1493T, X6426_SPLICE) had the same mutant frequency. To uncover the underlying reason for *MUC19* mutation being associated with clinical benefits of anti-PD-1 inhibitor treatment, we performed CD8 immunohistochemical staining. Compared to *MUC19* wild-type patients, *MUC19*-mutated patients exhibited more infiltration of CD8^+^ T cells (*P* = 0.0313) (Figures 5A,B). And patients with higher CD8^+^ T cells showed a significantly longer PFS (*P* = 0.00021) (Figure 5C).

### Association of *MUC19* Mutation With Immune Responses and Clinical Benefits of Anti-PD-1 Inhibitors in Public Datasets

*MUC19* is located on the long arm of chromosome 12 and encodes a member of the gel-forming mucin protein family which constitute the physical barrier, and protect epithelial cells from stress-induced damage (20, 21). *MUC19* is highly expressed in the corneal conjunctiva, lacrimal glands, and gastrointestinal glands, and is also expressed in the subtracheal glands (22). From the GeneCards website (https://www.genecards.org/), we identified that *MUC19* was similarly expressed in the lung, bone marrow, lymph node, thymus, and other immune system organs (Supplementary Figure 2A). It has been reported that MUC19 expression is involved in the pathogenesis of Sjogren syndrome and breast cancer; and breast cancer patients with higher MUC19 expression exhibited worse prognosis (23). In addition, *MUC19* mutation was found in inflammatory bowel disease, melanoma, colorectal adenocarcinoma, and esophageal squamous cell carcinoma (24–27). At present, what we understand regarding *MUC19* is limited, and the role of *MUC19* in lung cancer also remains unclear. This is the first study to explore and uncover the role of *MUC19* in lung cancer.

**Figure 6:**
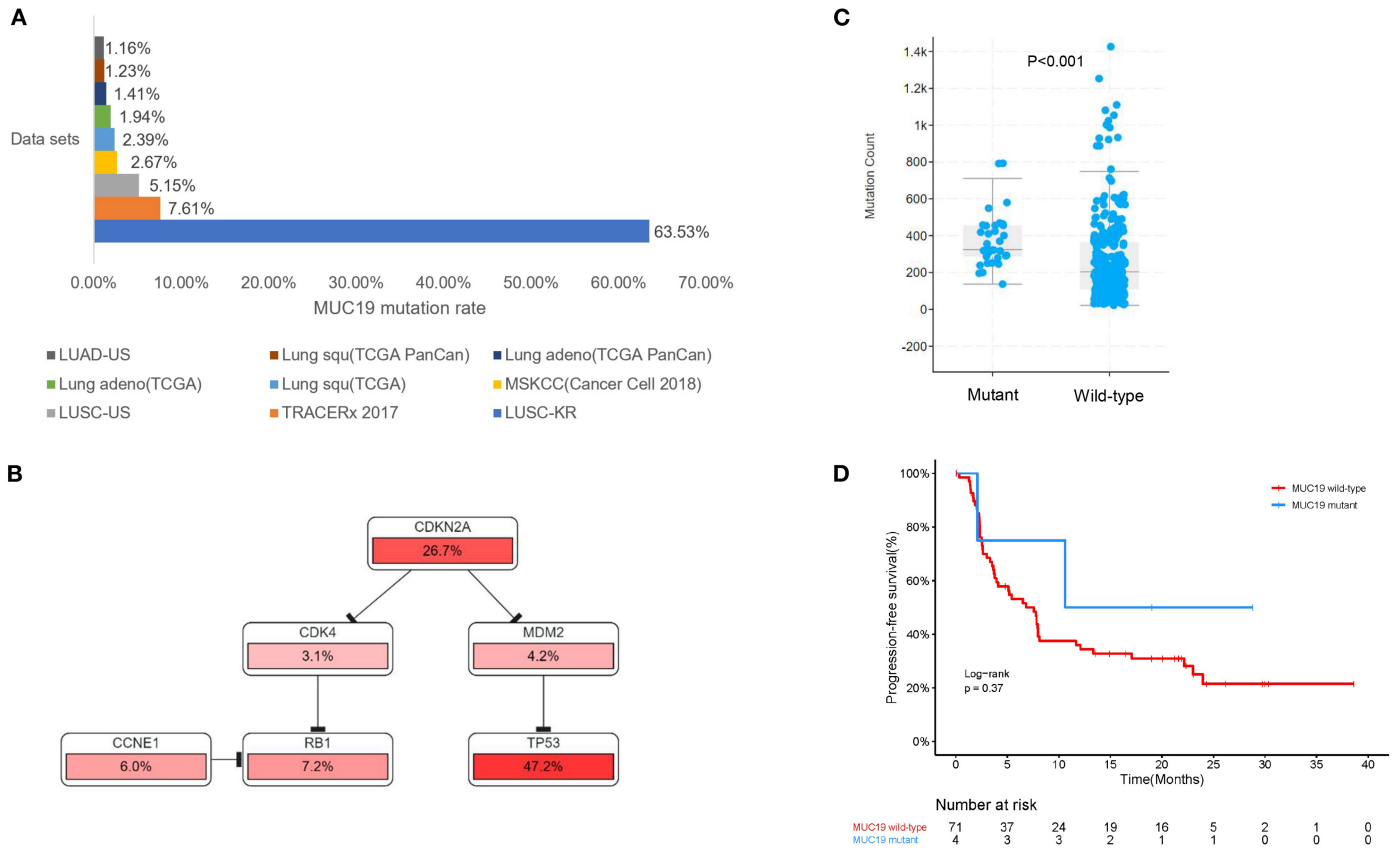
Association of *MUC19* mutation with immune responses and clinical benefits of anti-PD-1 inhibitors in public datasets. **(A)** Mutation rates identified using different public datasets. The LUSC-KR, LUSC-US, and LUAD-US data sets were downloaded from the International Cancer Genome Consortium website. The TRACERx 2017, MSKCC, Lung squ (TCGA), Lung adeno (TCGA), Lung adeno (TCGA PanCan), Lung squ (TCGA PanCan) were downloaded from the cBioPortal website. **(B)** Pathway mapper analysis of patients with or without *MUC19* mutation using the cBioPortal website. TP53-RB1 signaling pathway was the most frequently altered pathway. **(C)** Comparison of mutation count between patients with or without *MUC19* mutation in TRACERx 2017. **(D)** Kaplan-Meier curves of PFS comparing patients with mutated and wild-type *MUC19* in the MSKCC cohort.

Using the cBioPortal website, we downloaded all lung cancer datasets containing *MUC19* mutations (Figure 1B). These six studies included a total of 2,323 patients/2,672 samples, which included 1.5% Asian and 98.5% non-Asian populations. The mutation rate of *MUC19* was between 2 and 7% (Figure 6A). Surprisingly, from the International Cancer Genome Consortium Data Portal website, we found that the mutation rate of *MUC19* was 63.53% in a Korean cohort (LUSC-KR), which was very close to that of our study. However, the *MUC19* mutation rate in the LUSC-US and LUAD-US cohorts was < 6% (Figure 6A). The differential mutated genes between eastern (our cohort) and western (MSKCC cohort) people were shown in Figure 4B; compared to our cohort, the significantly higher mutation genes in MSKCC cohort were *KRAS, CTNND2, OBSCN*, and *DYNC2H1*, the significantly lower mutation genes were *NEFH, MUC19, ZNF141, PTH2, ZNF492, ADAMTSL4, MUC4, HOXB3, MUC5AC* and *MUC22*. Furthermore, we compared the difference of clinical characteristics between *MUC19* mutants versus *MUC19* wide-type patients in our cohort (Table 3), there were no statistical differences between them. As for the role of *MUC19* mutation on OS, we found that wild-type patients presented significantly lower OS compared to *MUC19*-mutated patients (*P* = 0.002) (Supplementary Figure 2B).

**Table 3:** Associations of *MUC19* mutation status with clinical characters in our cohort.

**Parameter**	***MUC19 wild-type***	***MUC19 mutant***	***P* value**
**Age**			1.000
<65	10	8	
≥ 65	9	6	
**Sex**			0.416
Male	13	12	
Female	6	2	
**BMI**			0.455
<24	12	11	
≥ 24	7	3	
**Performance status**			0.416
0–1	13	12	
≥2	6	2	
**Smoking status**			0.286
Former/Current	9	10	
Never	10	4	
**Histology**			0.854
Adenocarcinoma	10	6	
Squamous cell carcinoma	8	7	
Other	1	1	
**Stage**			0.257
III	4	6	
IV	15	8	
**Metastasis site**			
Lymph node (yes/no)	16/3	9/5	0.238
Lung (yes/no)	10/9	4/10	0.286
Bone (yes/no)	5/14	4/10	1.000
Liver (yes/no)	1/18	1/13	1.000
Brain (yes/no)	3/16	3/11	1.000
Adrenal (yes/no)	3/16	1/13	0.62
**PD-L1**			
<1%	4	4	0.716
≥1%	7	6	
Unknown	8	4	
**TMB**			0.363
<10 mut/Mb	17	10	
≥10 mut/Mb	2	4	

We further explored the role of *MUC19* mutations in immune responses. Gene Ontology (GO) annotation revealed that the *MUC19* gene is involved in innate immune response activating cell surface receptor signaling pathway (GO: 0002220). When using pathway mapper analysis on the cBioPortal website, the TP53-RB1 signaling pathway was the most frequently altered in the *MUC19* mutation group compared to the non-mutated group (Figure 6B). According to recent studies, *TP53* mutations could have a major impact on the lung tumor microenvironment (TME) and increase sensitivity to anti-PD-1 inhibitors in lung cancer (8). We also analyzed the mutation count in 100 patients/327 samples from another public dataset (TRACERx) through the cBioPortal website (28). *MUC19*-mutated patients had higher mutation counts than the non-mutated group (*P* < 0.001) (Figure 6C). It has been suggested that mutation count could reflect the whole exome mutational burden and that the mutation count of certain genes could be used as a new predictive marker to guide immunotherapy for NSCLC patients (29, 30).

More importantly, we validated our results in the MSKCC cohort containing 75 American lung cancer patients receiving immunotherapy and with *MUC19* mutation information. Although there was no significant difference, PFS of the *MUC19* mutation group was longer than that of the non-mutated patients (19.7 vs. 7.6 months, *P* = 0.413) (Figure 6D), which was consistent with our results.

## Discussion

Although the emergence of immunotherapy has dramatically changed treatment paradigms in NSCLC, only 20% of patients are able to benefit from immunotherapy (3). It is worth noting that some patients might suffer from significant immunotoxicity (4), and a large proportion of patients in China cannot afford them. Considering the low efficacy rate, immunotoxicity, and the drug cost, stratifying patients by specific biomarkers is essential. However, currently recommended biomarkers by National Comprehensive Cancer Network guidelines, such as PD-L1 and TMB, are not perfect biomarkers (31). Therefore, it is essential to discover novel biomarkers that are predictors of immunotherapy responses.

WES is a new method for identifying abnormalities in any gene. Compared to targeted gene panel sequencing, WES can discover abnormalities that have not been previously associated with any disease (32). Therefore, we chose WES to uncover novel gene mutations to identify immune checkpoint inhibitor responders in NSCLC. We identified a novel *MUC19* gene mutation from our data. In our study, both tumor tissue samples and matched control samples were tested, and patient matched control samples were used as negative controls. And none of these mutations detected in negative controls are included in our analysis, therefore, *MUC19* mutations found in our study are somatic mutations.

To our knowledge, this is the first study characterizing *MUC19* in lung cancer. We found that the mutation rate of *MUC19* in lung cancer was higher in Asian patients than in non-Asian patients (LUSC-KR 63.53% vs. LUSC-US 5.15%; LUSC-US 5.15% vs. LUAD-US 1.16%). Consistent with other studies (33, 34), we also found the different mutation rate of KRAS influenced by ethnicity in our study (Figure 4B). In addition, environmental factors could also affect gene mutations. Bacterial infection (*F. nucleatum* and *B. fragilis*) led to gene mutations (35), tobacco exposure had an effect on intestinal microbiome and could also produce new gene mutations (36, 37). More interestingly, bacterial infection (*Streptococcus pneumoniae*, nontypeable *Haemophilus influenzae*) upregulated MUC19 expression (38). Hence, the effect of microbiome and tobacco exposure on *MUC19* mutation needs further research.

**Figure 7:**
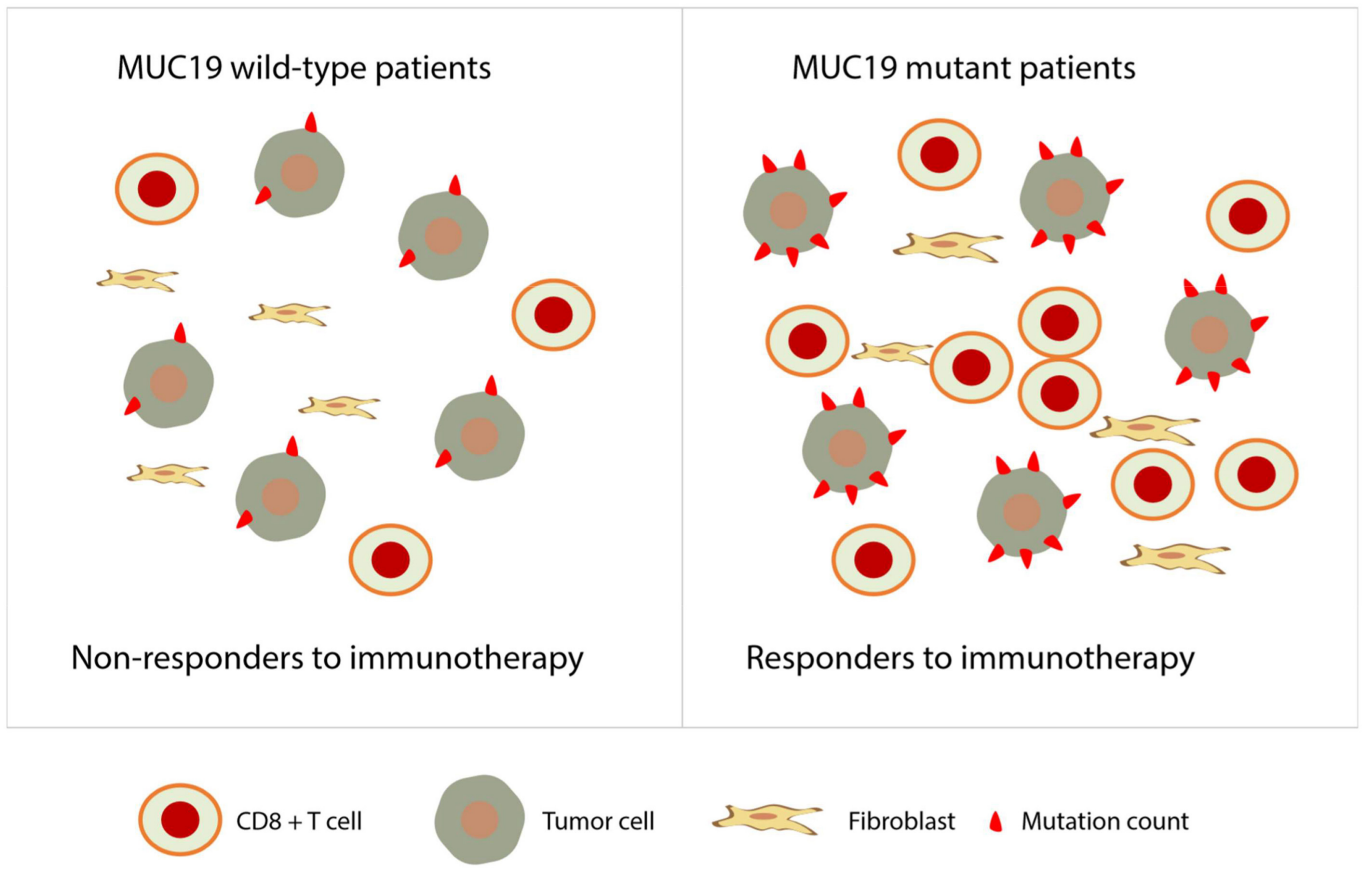
Proposed role of *MUC19* mutation in predicting clinical benefits of immunotherapy. *MUC19*-mutated NSCLC patients are associated with more infiltration of CD8^+^ T cells and higher mutation count, therefore, they are more likely to be responders to immunotherapy.

Remarkably, for the first time, we uncovered a predictive role of *MUC19* mutation in NSCLC patients receiving anti-PD-1 inhibitors. We aimed to understand the underlying mechanism behind this phenomenon. First, we detected infiltration of CD8^+^ T cells in the TME. We found an association of *MUC19* mutation with more CD8^+^ T cells (Figure 7), which suggests a “hot” TME (39). Second, we searched public datasets to uncover the inner connections and causality of *MUC19* mutations with immune responses. Both GO annotation and cBioPortal pathway mapper analysis indicated the involvement of *MUC19* mutation in immune responses. Lastly, but most importantly, we validated our results in the MSKCC cohort. Compared to wild-type patients, *MUC19*-mutated patients showed a trend for increased PFS, although this was not statistically significant, likely owing to the small number of patients studied. The MSKCC group is an American cohort, so the mutation rate was relatively low in this cohort. Therefore, it was difficult to observe a predictive role for *MUC19* mutations in this cohort. In the future, larger studies are needed to validate our results, especially in Asian patients. In addition to *MUC19* mutation, we also found that gene mutations such as those involving *PKD1L2* and *OTOGL* were poor prognostic factors for immunotherapy. Considering the low numbers of mutation-positive patients, we did not analyze the related information in public datasets. Additional studies are needed to confirm these results.

Although TMB is recommended by the Food and Drug Administration as a new predictive biomarker for patients with unresectable or metastatic solid tumors receiving pembrolizumab, new KETNOTE021 data showed no association of TMB with the efficacy of pembrolizumab plus carboplatin and pemetrexed (40). In our cohort, 75.8% of patients received a combination of immunotherapy and chemotherapy. Therefore, it is not difficult to understand that there were no differences between the two groups.

Taken together, the originality of our work relies on the fact that we uncovered a novel role of *MUC19* mutation in predicting the efficacy of anti-PD-1 inhibitors. Furthermore, we analyzed the association of *MUC19* status with infiltration by CD8^+^ T cells in the TME. As the sample size in our study was small and represented only a single center investigation, we validated our results using public datasets. Although not perfect, we have discovered potential influencing factors surrounding the clinical benefits of anti-PD-1 inhibitors. Future studies should aim to characterize the role of *MUC19* mutation in mediating cancer immune responses, and large-scale prospective studies will be required to validate our results.

## Data Availability Statement

The whole exome sequencing data has been deposited into a publicly accessible repository: http://db.cngb.org/cnsa/project/CNP0001349/reviewlink/.

## Ethics Statement

The studies involving human participants were reviewed and approved by Medical Ethics Committee of Jinling Hospital. The patients/participants provided their written informed consent to participate in this study. Written informed consent was obtained from the individual(s) for the publication of any potentially identifiable images or data included in this article.

## Author Contributions

YSo, TL, and DL conceived and designed the experiments. LZ, LH, and QX drafted the manuscript and produced the figures. LZ, YL, ZW, and HH were responsible for data collection and analysis. LH, QX, YSh, and PZ performed statistical analyses and edited references. YSo, TL, and DL revised the manuscript. All authors have approved the final version of the manuscript.

## Conflict of Interest

YSh was employed by the company Geneseeq Technology Inc. The remaining authors declare that the research was conducted in the absence of any commercial or financial relationships that could be construed as a potential conflict of interest.

## Abbreviations

BMI: body mass index
DCB: durable clinical benefit
ECOG: Eastern Cooperative Oncology Group
GO: Gene Ontology
MSKCC: Memorial Sloan-Kettering Cancer Center
NDB: no durable benefit
NSCLC: non-small cell lung cancer
OS: overall survival
PFS: progression-free survival
TMB: tumor mutational burden
TME: tumor microenvironment
WES: whole exome sequencing.
