# Supplementary figures and images for: Association of MUC19 Mutation With Clinical Benefits of Anti-PD-1 Inhibitors in Non-small Cell Lung Cancer
Source: Front Oncol. 2021 Mar 22;11:596542. doi: 10.3389/fonc.2021.596542 (PMC8019943; doi:10.3389/fonc.2021.596542)

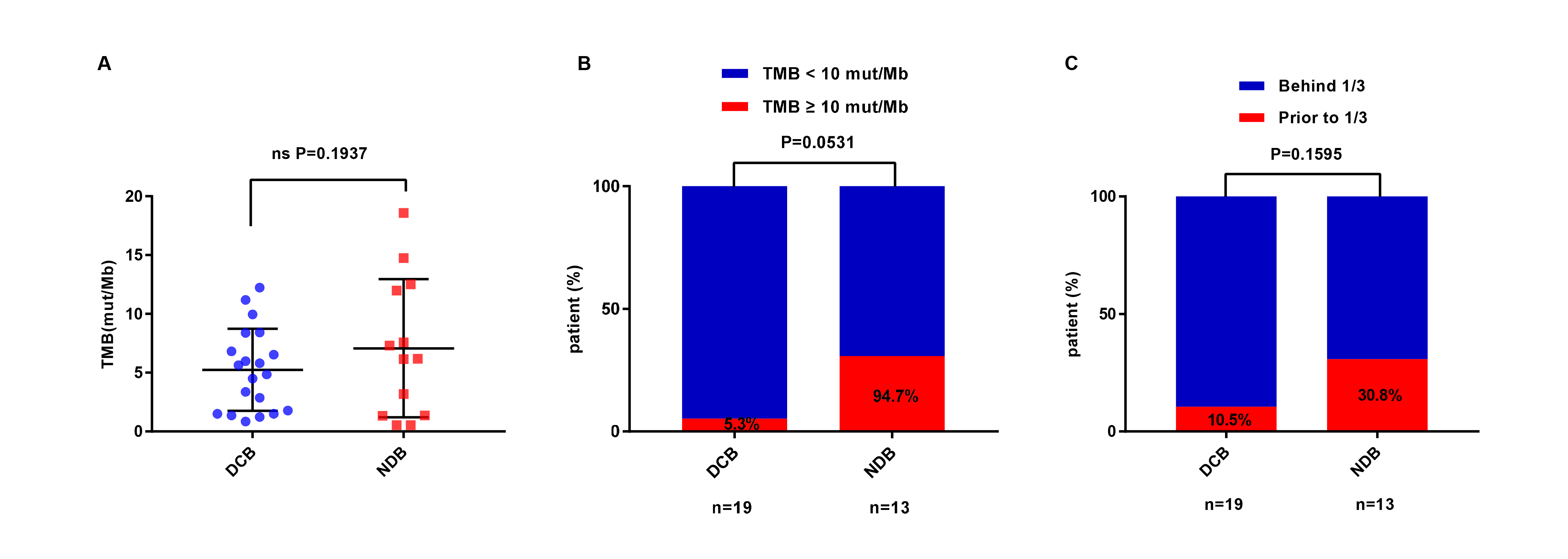

Supplement: Supplementary file 1 [file Image_1.TIF]

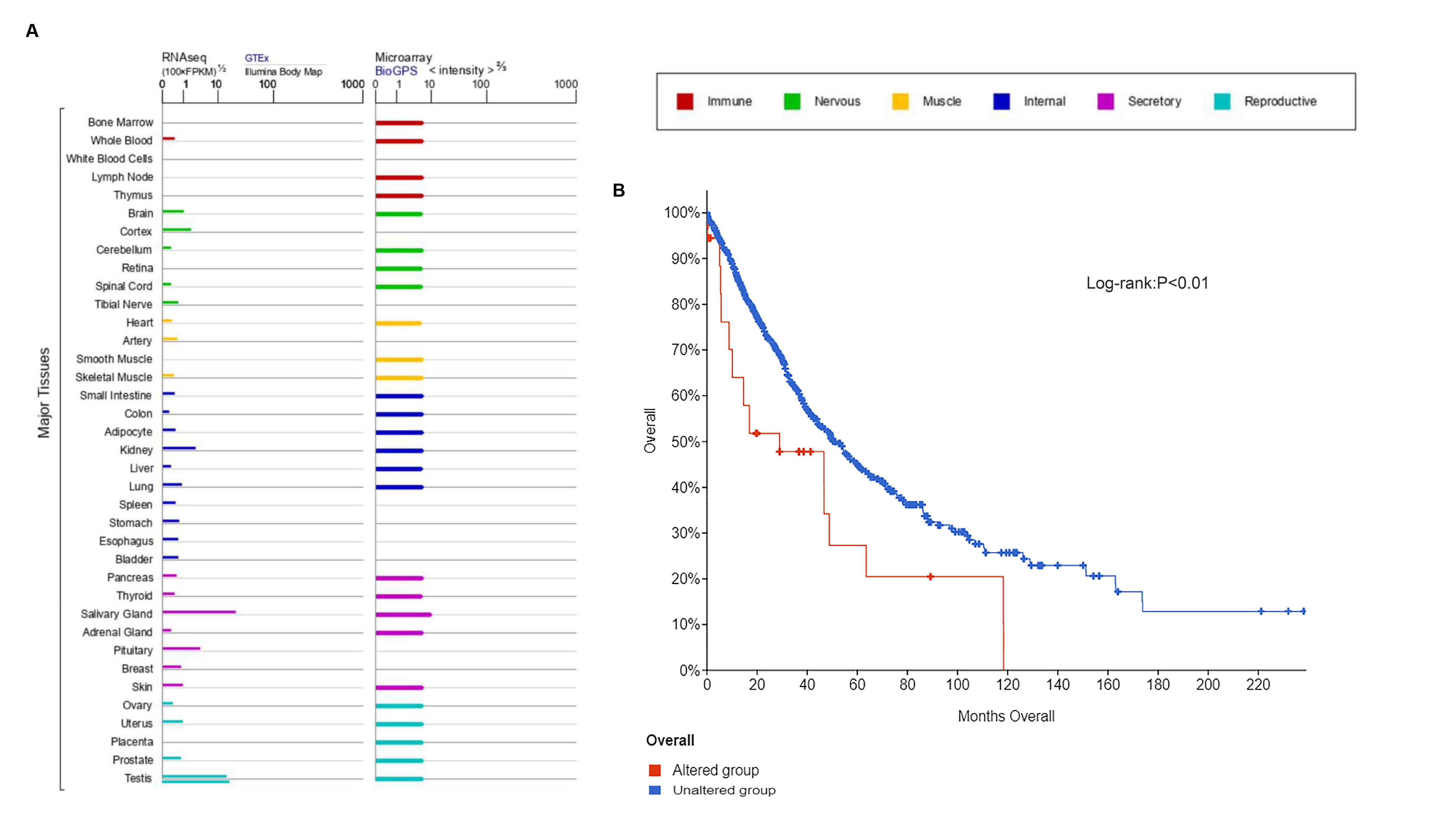

Supplement: Supplementary file 2 [file Image_2.TIF]
