# Supplementary material for: Association of MUC19 Mutation With Clinical Benefits of Anti-PD-1 Inhibitors in Non-small Cell Lung Cancer
Source: Front Oncol. 2021 Mar 22;11:596542. doi: 10.3389/fonc.2021.596542 (PMC8019943; doi:10.3389/fonc.2021.596542)
Supplement: Supplementary file 3 [file Data_Sheet_1.docx]

**Table S1. Baseline clinical characteristics of NSCLC patients in MSKCC cohort.**

| **Characteristics** | **Total(N=75)** | **%** |
| --- | --- | --- |
| **Age(years)，median range**  ＜65  ≥65  **Sex**  Male  Female  **Performance status**  0  1 | **66**  **39**  **36**  **37**  **38**  **30**  **45** | **(42-87)**  **52**  **48**    **49.3**  **50.7**    **40**  **60** |
| **Smoking status**  Former/Current  Never  **Histology**  Squamous  Non-squamous  **Clinical benefit**  DCB  NDB  **Stage**  III  IV | **60**  **15**  **16**  **59**  **37**  **38**  **9**  **66** | **80**  **20**    **21.3**  **78.7**    **49.3**  **50.7**    **12**  **88** |

**Table S2. Individual gene mutations associated with Anti-PD-1 inhibitor’s response in our cohort.**

| Gene | HR | 95%CI for HR | Log-rank P value |
| --- | --- | --- | --- |
| OTOGL | 17.6 | 2.4-127.7 | ＜0.001 |
| KIAA1217 | 7.2 | 1.7-29.5 | 0.001 |
| GSE1 | 5.1 | 1.4-18.9 | 0.006 |
| PKD1L2 | 4.5 | 1.3-15.1 | 0.006 |
| FSIP2 | 4.9 | 1.2-18.5 | 0.009 |
| FNDC1 | 4.7 | 1.2-17.5 | 0.01 |
| SRRT | 3.4 | 1.1-10.4 | 0.021 |
| SCN5A | 4.2 | 1.1-16.1 | 0.022 |
| MUC19 | 0.34 | 0.1-0.9 | 0.026 |
| LRRK2 | 4.7 | 1.0-22.2 | 0.028 |
| TOPAZ1 | 3.8 | 1.0-14.7 | 0.033 |

**Figure S1. Comparison of TMB between DCB and NDB patients in our cohort.**

(**A**) TMB in DCB (blue, n = 20) versus NDB (red, n = 13) patients. (**B**) The proportion of high TMB (>=10mut/Mb) and low TMB (<10mut/Mb) patients in DCB and NDB groups. (**C**) The proportion of high TMB (prior 1/3) and low TMB (behind 1/3) patients in DCB and NDB groups.

**Figure S2: Role of MUC19 in lung cancer.** (**A**) Expression profiles of MUC19 in human tissues from GeneCards website. (**B**) Kaplan-Meier curves of OS comparing *MUC19*-mutated and wild-type patients (mPFS 28.94 versus 49.80 months, P = 2.255e-3).
